# High-performance flexible thermoelectric modules based on high crystal quality printed TiS_2_/hexylamine

**DOI:** 10.1080/14686996.2021.1978802

**Published:** 2021-11-24

**Authors:** Stéphane Jacob, Bruno Delatouche, Daniel Péré, Zia Ullah Khan, Marc Jacques Ledoux, Xavier Crispin, Radoslaw Chmielowski

**Affiliations:** a Department of Advanced Materials, IMRA Europe S.A.S., Sophia Antipolis, France; b Laboratory of Organic Electronics, Department of Science and Technology, Linköping University, Norrköping, Sweden; c Institut de Chimie et Procédés Pour l’Energie, l’Environnement et la Santé (ICPEES), UMR 7515 CNRS/Université de Strasbourg, Schiltigheim, France

**Keywords:** TiS_2_/hexylamine, exfoliation, thin films, printed thermoelectrics, layered structure, 50 Energy Materials, 105 Low-Dimension (1D/2D) materials, 210 Thermoelectronics / Thermal transport / insulators, 206 Energy conversion / transport / storage / recovery

## Abstract

Printed electronics implies the use of low-cost, scalable, printing technologies to fabricate electronic devices and circuits on flexible substrates, such as paper or plastics. The development of this new electronic is currently expanding because of the emergence of the internet-of-everything. Although lot of attention has been paid to functional inks based on organic semiconductors, another class of inks is based on nanoparticles obtained from exfoliated 2D materials, such as graphene and metal sulfides. The ultimate scientific and technological challenge is to find a strategy where the exfoliated nanoparticle flakes in the inks can, after solvent evaporation, form a solid which displays performances equal to the single crystal of the 2D material. In this context, a printed layer, formed from an ink composed of nano-flakes of TiS_2_ intercalated with hexylamine, which displays thermoelectric properties superior to organic intercalated TiS_2_ single crystals, is demonstrated for the first time. The choice of the fraction of exfoliated nano-flakes appears to be a key to the forming of a new self-organized layered material by solvent evaporation. The printed layer is an efficient n-type thermoelectric material which complements the p-type printable organic semiconductors The thermoelectric power factor of the printed TiS_2_/hexylamine thin films reach record values of 1460 µW m^−1^ K^−2^ at 430 K, this is considerably higher than the high value of 900 µW m^−1^ K^−2^ at 300 K reported for a single crystal. A printed thermoelectric generator based on eight legs of TiS_2_ confirms the high-power factor values by generating a power density of 16.0 W m^−2^ at ΔT = 40 K.

## Graphical abstract

**Figure:**
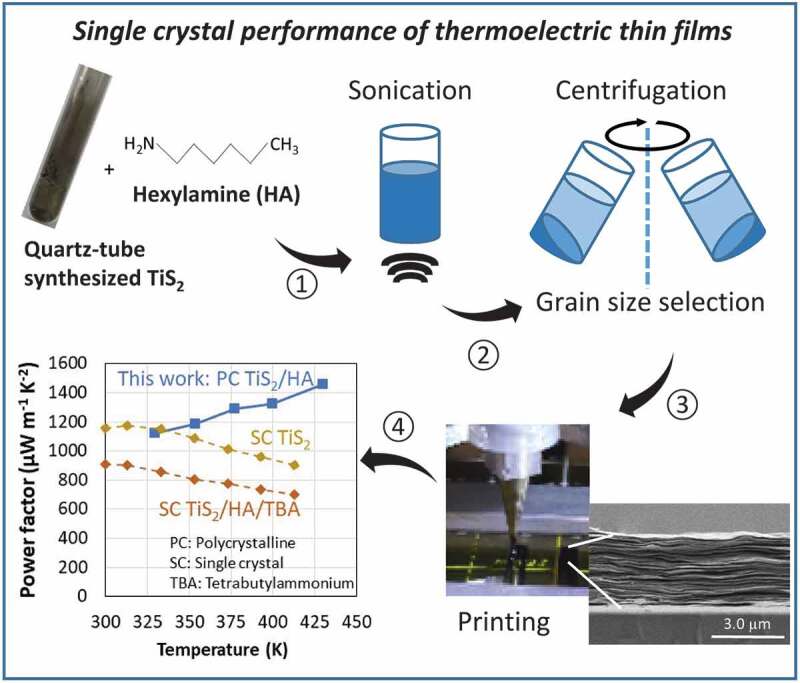

## 1. Introduction

The interest for printable and flexible thermoelectric devices has increased in recent years as a result of many potential applications such as wearable energy harvesting [1–7], the powering of wireless sensors for the internet-of-everything [8,9], ultra-sensitive thermopiles [10], etc. Of these different materials, p-type printed thermoelectric devices based on organic semiconductors have achieved reasonable performances [11,12]. zTs values in the range of 0.3–0.4 at room temperature were obtained with the polymer poly(3,4-ethylenedioxy-thiophene):polystyrene sulfonic acid (PEDOT:PSS) by tuning its level of doping [13,14]. Besides its low cost, the advantage of such a polymer is its intrinsic low thermal conductivity (~0.3 W m^−1^ K^−1^ for the through-plane value and below 1 W m^−1^ K^−1^ for the in-plane value) [15] and its high-power factor (PF) of approximately 470 µW m^−1^ K^−2^. Another class of printable compounds based on exfoliated 2D materials was considered. The reason is their low dimensionality, a feature favorable for high performances [16]. Exfoliated organic compounds such as graphene have not been able to compete with PEDOT:PSS because of its intrinsic high thermal conductivity [17]. On the other hand n-type exfoliated inorganic thermoelectric materials based on metal chalcogenide compounds were considered as promising, due to their low cost, environmental friendliness and the high reported power factor of their bulk form [18–20]. One of these compounds, TiS_2_ was recently used to prepare new n-type hybrid TiS_2_/organic superlattice materials as efficient n-type counterparts to the printed p-type PEDOT:PSS films. Hence, this choice of n- and p-type thermoelectric materials is today the most promising for the fabrication of efficient thermoelectric generators based on printing processes without high-temperature treatments, i.e. enabling a range of flexible substrates such as paper and plastics which are crucial in packaging and their further implementation in the internet-of-everything [21].

The n-type hybrid TiS_2_/organic superlattice materials were synthesized firstly by an electrochemical intercalation of hexylammonium (HA^+^) cation in single crystals of TiS_2_ and co-intercalation of the dimethyl sulfoxide (DMSO) solvent [22]. Part of the solvent was then exchanged with H_2_O to yield TiS_2_[(HA)_x_(H_2_O)_y_(DMSO)_z_]. In this compound the thermal conductivity of the composite layered material was reduced to 0.69 W m^−1^ K^−1^ at room temperature. Its in-plane power factor was furthermore measured at 450 µW m^−1^ K^−2^ and the zT was calculated to be 0.28 at 373 K, which is 3 times higher than TiS_2_. It is known both for organic and inorganic materials, that tuning the charge carrier concentration enables the optimization of the power factor because the Seebeck coefficient and electrical conductivity evolve in opposite directions with charge carrier concentration [23,24]. This was implemented by replacing the solvents co-intercalated with HA^+^ by tetrabutylammonium (TBA) and the power factor was then increased to 900 µW m^−1^ K^−2^ at room temperature (RT) [25]. TBA has a higher boiling point and higher molecular weight than HA^+^. In this new system, the thermal conductivity remained quite low and in consequence an increase of zTs to 0.31 at 373 K and 0.33 at 430 K was observed.

**Figure 1.**
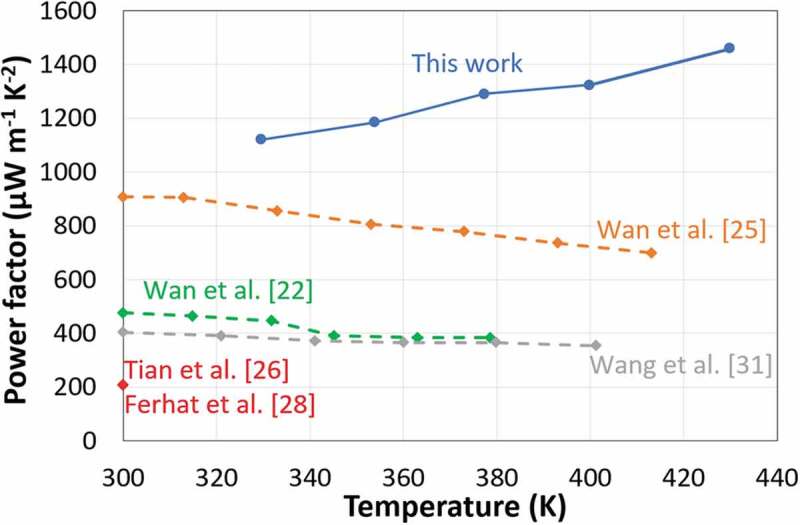
The power factor of this work vs. the previously reported values. The power factor of the intercalated TiS_2_ single crystal is represented by a dashed line; while the intercalated TiS_2_ polycrystalline is identified with a continuous line, with in addition the red point

The costly process relying on TiS_2_ single crystals was replaced and the electrochemical intercalation process was modified. Hence polycrystalline TiS_2_ powder was prepared and intercalated with HA by mechanical treatment [26,27]. The resulting compound was further processed in solution to yield thin films with a power factor of about 210 µW m^−1^ K^−2^ at RT. More recently, similar power factor of about 210 µWm^−1^K^−2^ were obtained on TiS_2_[HA]_x_ nanocomposite printed on a paper substrate [28]. Although the power factor was more than 4 times lower than the intercalated TiS_2_ single crystal, these studies demonstrated the potential of solution process for fabricating 2D thermoelectric inorganic nanomaterial layers. This technology based on hybrid intercalated TiS_2_ materials was also the subject of patent applications [29,30]. Further studies to increase the power factor, a composite of polycrystalline TiS_2_ and fullerene was carried out [31]. In this system assembling C_60_ molecules on the surface of the TiS_2_ nanolayers led to a PF which was doubled compared to the paper cited above. However, it still lies far below the performance achieved with the intercalated single crystal. We sum up the results of all those studies on TiS_2_ in Figure 1.

In this study, we show that it is possible to intercalate HA in polycrystalline TiS_2_ to get power factors of about 1400 µW m^−1^ K^−2^ at 420 K, i.e. higher than the intercalated TiS_2_ single crystal (Figure 1). Moreover, we demonstrate a low-cost manufacturing process based on the deposition of high performance TiS_2_ films from a solution process at low temperature. This work paves the way for a possible replication of multiple patterns of p- and n-legs by printing technologies; which is required to reach the thermo-voltage up to useful values in electronics (order of 1 volt).

## 2. Experimental section

### 2.1. Synthesis of TiS_2_ powder

The TiS_2_ powder was synthesized by a solid-state reaction [32] from stoichiometric amounts of Ti (Goodfellow) and S (Sigma Aldrich) powders. The precursors were mixed in a mortar, poured into a quartz ampoule that was sealed under vacuum, placed in a vertical furnace and subsequently heated at 700°C for 24 h. The mixture was finally cooled to RT. The recovered compound was ground in a mortar and analyzed by X-ray diffraction (XRD). The pattern corresponded to hexagonal TiS_2_ with space group P-3m1 (164) [33]. Some sublimated sulfur was found on the inner wall which could indicate a slightly stoichiometric deviation towards S-rich synthesis conditions. Previous reports have shown that the excess of Ti can intercalate in the Van der Waals gap of TiS_2_ greatly improving the thermoelectric properties of the compound [19].

### 2.2. Intercalation of hexylamine in TiS_2_

The intercalation of hexylamine in TiS_2_ as well as the ink formulation process are described and discussed in the core of this paper.

### 2.3. Printing of the films

The as-prepared ink was poured in a cartridge, which was installed in a dispenser and the dispenser mounted on the V-One printer from Voltera (Canada). The ink was then dispensed via a nozzle onto the desired substrate. The movement of the dispenser in 3 directions was computer-controlled that allowed to print AutoCAD draw patterns. The samples printed on a glass substrate or Kapton® tape had a size of 2 × 15 mm^2^ and a thickness of about 2.5 µm.

### 2.4. Structural characterizations

The scanning electron microscopy (SEM) investigations were carried out on a Hitachi (Japan) S-4700 equipped with a field emission gun. The XRD patterns were collected at room temperature on thin films printed on glass substrates using a Bruker (Germany) D8 diffractometer equipped with a copper anode and a point detector.

### 2.5. Thermoelectric characterization of the films

The electrical resistivity (ρ) and the Seebeck coefficient (S) of the films deposited on glass were directly measured as a function of temperature under a helium atmosphere on a Linseis (Germany) LSR-3 setup. The electrical conductivity (σ) was then calculated via σ = 1/ρ. The thickness of the films was measured by SEM analysis of their cross-sections. The samples for these characterizations were deposited on glass substrates and had 4 × 20 mm^2^ in size and about 2.5 µm in thickness. The thermal conductivity (κ) was measured on films which were deposited by Blade casting on chips supplied free of charge by Linseis. The chips were then sent to Linseis and their thermal conductivity was measured using the Thin Film Analyzer of the company [34]. The figure of merit zT was then calculated via zT = (S^2^*σ*T)/κ. Hall effects measurements were made on an Ecopia (Korea) HMS-5500 material mounted with an AHT55T5 high temperature magnet. The voltage generated by the TEG stacks was measured using a home-made set up based on a Peltier device as hot source and ambient air as cold source.

## 3. Results and discussion

### 3.1. Process for the fabrication of the TiS_2_/HA thin films

**Figure 2.**
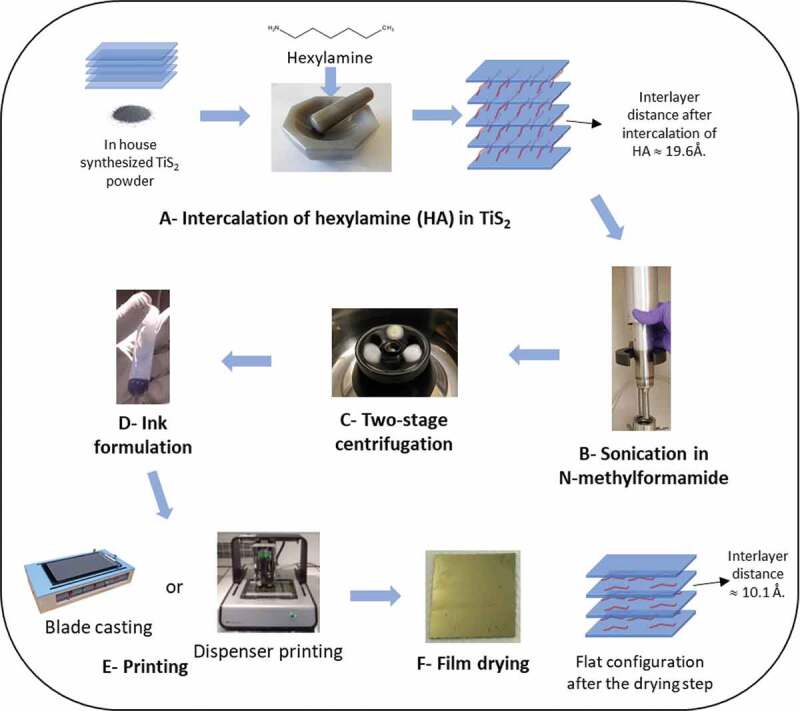
Process for the preparation of Hybrid TiS_2_/HA thin films. (a) Intercalation of TiS_2_/hexylamine 1/3 molar ratio by mixing and grinding of the chemicals. (b) Dispersion of the slurry in N-methylformamide and sonication of the suspension. (c) Centrifugation at 1000 rpm of the recovered material. (d) Supernatant recovered and centrifuged at 9000 rpm. Sediment separated from the supernatant and further used as ink. (e) Deposition of the film either by blade casting or dispenser printing. (f) Film drying at 90°C for 30 minutes

The different steps of the fabrication process are shown in Figure 2. Steps a and b are similar to those published by Tian et al. [26] However, it is worth mentioning that the quality of the TiS_2_ powder seems to be an important parameter. The formulation process carried out with a commercially available TiS_2_ powder always led to thin films with much lower thermoelectric performances. Scanning electron microscopy observations revealed a different crystal morphology between the two samples and a narrower particle size distribution for the self-made TiS_2_ that led to films with a perfect layered structure. The commercially available TiS_2_ did not lead to such a continuous layered structure as shown in Note S1 and Figure S1 (Supporting Information), which could explain the lower performances of its films reproduced in Figure S2 (Supporting Information). The formulation process presented is performed on TiS_2_ powder which is not exposed to air and the entire formulation process is performed in an argon atmosphere with a O_2_ level below 1 ppm. In step c, a two-stage centrifugation is applied. First, a centrifugation speed of 1000 rpm is used to remove the large particles (Figure S3 and S4, Supporting Information). Then, the supernatant is recovered and instead of using it directly as precursor for the films, it is centrifuged in a second stage at a higher speed of 9000 rpm. The sediment obtained is collected and used as the ink for further preparation of the films. The proposed formulation process is more adapted to a future scale-up using ink as a precursor of the films rather than evaporating the solvent of a supernatant to create the films on substrates. Another notable feature of this method is that the two-stage centrifugation removes the big particles from the ink as demonstrated by the SEM images shown in Note S2 and Figure S3 (Supporting Information). The ink could be then deposited by various printing technologies such as blade casting or dispenser printing. The final films were heated at 90°C for 30 minutes to remove the excess of HA and N-methylformamide.

### 3.2. Investigations on the intercalation process

**Figure 3.**
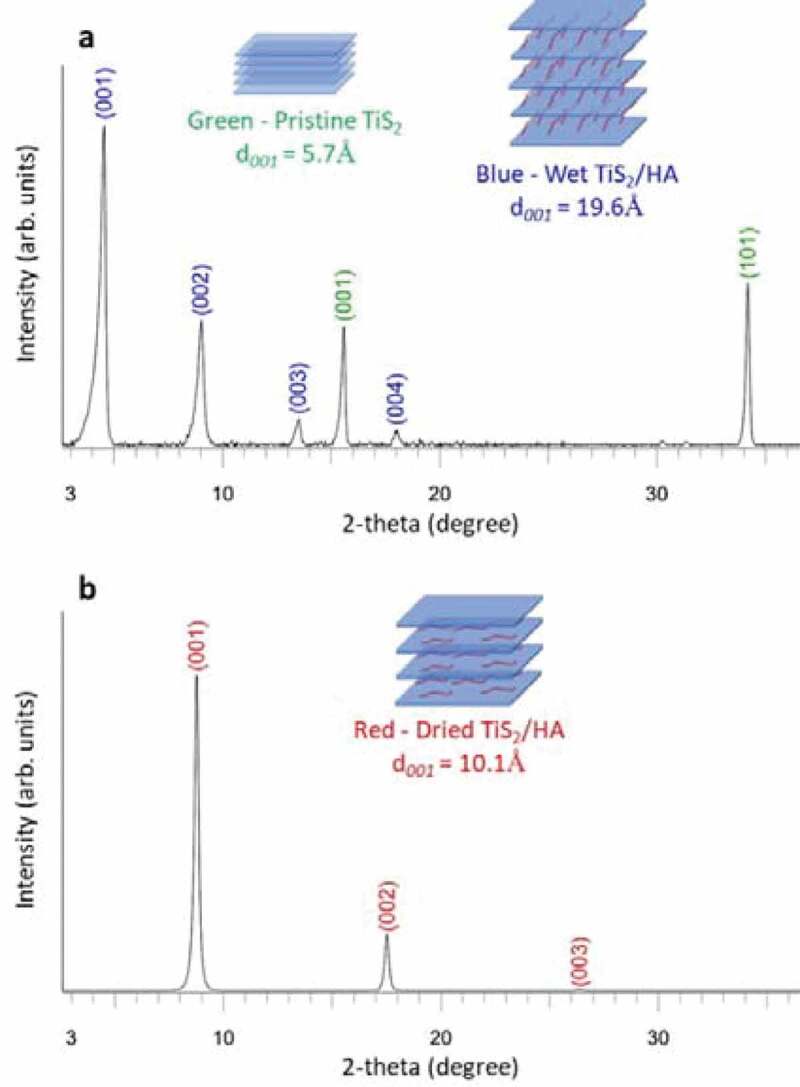
X-ray diffraction patterns collected at different stages of the fabrication process. (a) XRD pattern of the slurry obtained by grinding TiS_2_ and HA with a 1:3 molar ratio. (b) XRD pattern of the TiS_2_/HA dried film

The X-ray diffraction (XRD) patterns collected after various steps of the formulation process are shown in Figure 3. After mixing the hexylamine with TiS_2_ (Figure 2(a)) the XRD detects two crystal phases. The pristine TiS_2_ is identified by its two main peaks, namely (001) and (101) located at 15.6**°** and 34.2**°** in 2-theta, respectively. The presence of the hybrid TiS_2_/HA material is detected by a series of (00 ***l***) peaks with ***l*** = 1, 2, 3 and 4 (Figure 3(a)). This series of peaks indicates that the hexylamine has been intercalated between the (001) planes of TiS_2._

The main peak of the hybrid material located at 4.5° corresponds to a distance of 19.6 Å. According to A. Weiss et al. [35] the intercalation of alkylamines C_n_H_2n+1_NH_2_ (4 < n < 17) between the TiS_2_ layers leads to an increase of interlayer distance and the organic molecules organize with a tilt angle compared to the TiS_2_ plane that depends on their length. For hexylamine this angle is about 56° [35]. During the drying step (Figure 2(f)) at elevated temperature the arrangement of the organic molecules inside the hybrid TiS_2_/HA material changes. The main (00 ***l***) peak located at 4.5° in 2-theta is shifted towards a higher 2-theta angle of 8.7°. This corresponds to the modification of the interlayer distance in the hybrid material from 19.6 Å to 10.1 Å (Figure 3(b)). This modification of the interlayer distance corresponds to a reorganization of the organic molecules between the TiS_2_ sheets. After the drying step the intercalated HA molecules form a monolayer sandwiched in the Van der Waals gap of TiS_2_ and lie parallel to the TiS_2_ sheets [26].

### 3.3. Microstructure characterization of the thin films

**Figure 4.**
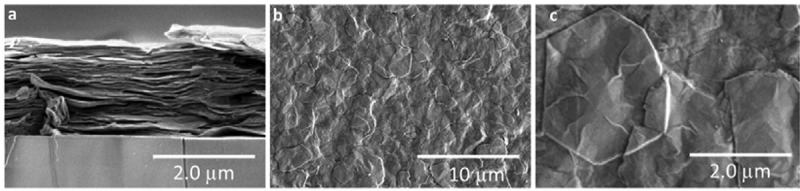
SEM images of a film prepared by blade casting. (a) Cross-section view. (b) Top view at low magnification showing that the film is composed of 2D thin flakes, (c) Top view at high magnification showing a characteristic hexagonal shape of TiS_2_ flake

SEM micrographs of a printed TiS_2_ film on a glass substrate are shown in Figure 4. The cross-section view reveals the layered structure based on the hybrid inorganic-organic TiS_2_/HA. Hence, even when processed from solution, the evaporation of the suspension of TiS_2_ particles leads to a spontaneous alignment of the intercalated TiS_2_ sheets parallel to the substrate. Such a well-organized alignment has been attributed to the highly anisotropic character of the TiS_2_/HA nanosheets and to the forces which are exerted by surface tension while the solvent evaporates [27]. Furthermore, few typical hexagonal flakes of the TiS_2_ material are visible at low and high magnifications top-view SEM images (Figure 4(b and c)) despite the energetic mechanical process of exfoliation. The fact that it is possible to see the structure underneath the TiS_2_ flakes indicates their extremely thin nature. The two-stage centrifugation process allowed the selection of TiS_2_ flakes with a lateral size varying from a few hundred nanometers for the smallest up to 3000 nm for the largest.

**Figure 5.**
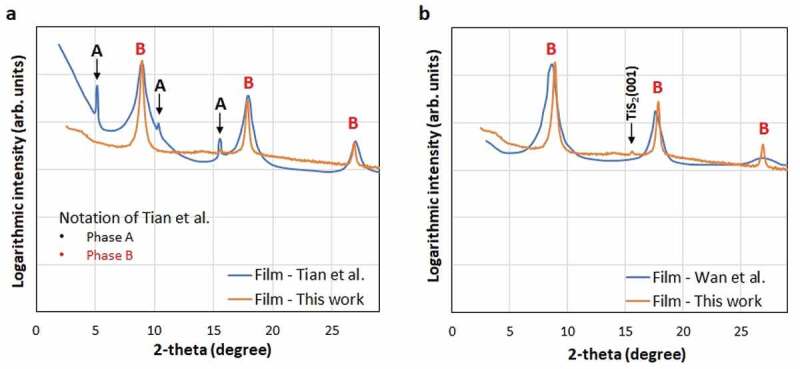
Comparison of the X-ray diffraction diagrams collected on the TiS_2_/HA films in this publication and (a) Tian et al. [26] and (b) Wan et al. [27]. Redrawn with the permissions of The Royal Society of Chemistry and Elsevier

Further analysis of the thin film microstructure suggests a good agreement between the observed SEM observations and the collected XRD patterns. Indeed, the TiS_2_/HA grains lying parallel to the substrate (Figure 4(a)) cause a strong diffraction from the (00 ***l***) planes (Figure 5). It is worth mentioning that the XRD pattern collected indicates a superior phase purity and crystallinity of thin films in comparison to previous reports. In particularly, Tian et al. [26] observed two intercalated phases called A and B; while in this work only the intercalated phase B is detected, which corresponds to dried hybrid TiS_2_/HA crystals. The film in these experiments has therefore a high crystal phase purity. Furthermore, the crystallinity of TiS_2_/HA crystals in this film is better as the full width at half maximum (FWHM) of the peaks is narrower than for the corresponding peaks of previously reported films (e.g. the peak at 8.7° has a FWHM of 0.233° in our film and 1.06° in the film of Tian et al.) (Figure 5(a)). Similar trend is observed in comparison to Wan et al. [27] (Figure 5(b)) where narrow FWHM on B1, B2 and B3 peaks indicate a high crystallinity in our films (e.g. FWHM of B1 is 0.233° while it is 1.25° in the previous study). Finally, very recently, Gu et al. [36] observed a significant increase of the thermoelectric power factor on a mechanically exfoliated and restacked at high-temperature bulk pristine TiS_2_. In this independent study Gu et al. also demonstrate that the crystal quality seems to be the key parameter in achieving the high thermoelectric properties in 2D based materials.

### 3.4. Thermoelectric properties of the thin films

In the past, several theoretical and experimental investigations have been carried to explain transport properties of Ti_1+x_S_2_ systems [19,20,37–39]. Authors agree that a perfect TiS_2_ crystal is a narrow band gap semiconductor that tends to have a (semi)metallic behavior because of the titanium interstitial defects (Ti_i_) located in the van der Waals gaps. It means, that for low concentrations of Ti_i_ the material keeps its semiconducting behavior with a relatively high Seebeck coefficient while for high Ti_i_ concentrations the material behaves like a semimetal with a low Seebeck coefficient. Experimental data confirms the sensitivity of the Seebeck coefficient to small excess of Ti (about 1% excess of Ti leads to a decrease of the Seebeck coefficient by more than half its value [19,39]). Hence, reports in the literature might include a slight difference in the purity of the TiS_2_, which is difficult to detect but may significantly affect the electrical conductivity and the Seebeck coefficient of the TiS_2_ before the hexylamine intercalation.

**Figure 6.**
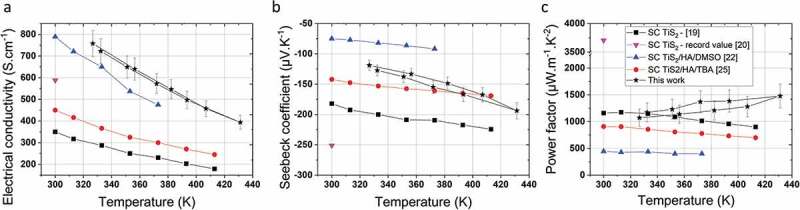
In-plane thermoelectric properties of a film from this work in comparison to previous reports on single crystal TiS_2_ (SC TiS_2_) [19,20], single crystal TiS_2_ intercalated with hexylamine and DMSO (SC TiS_2_/HA/DMSO) and single crystal TiS_2_ intercalated with hexylamine and tetrabutylammonium (SC TiS_2_/HA/TBA) [22,25]. Temperature dependence of the electrical resistivity (a), the Seebeck coefficient (b) and the power factor (c).(The measurements were carried out for a heating and cooling with an accuracy for the Seebeck coefficient of ± 7%, for the electrical conductivity is ± 8%, for the power factor is ± 15%)

The electrical conductivity and the Seebeck coefficient of the printed TiS_2_/HA are 740 S cm^−1^ and −123 µV K^−1^ at 330 K, respectively. These values are superior to the one reported for an intercalated TiS_2_/HA/DMSO single crystal (650 S cm^−1^ and −78 µV K^−1^ at 330 K) [25]. Remarkably, the power factor of the micrometer thick films formed by the printing process increases from 1120 µW m^−1^ K^−2^ at 330 K to 1460 µW m^−1^ K^−2^ at 430 K at the best. The comparison of the power factor at 420 K demonstrates superior thermoelectric performances (i.e. 1400 µW m^−1^ K^−2^) in comparison to hybrid TiS_2_/HA single crystals (700 µW m^−1^ K^−2^) reported by Wan et al. and other solution-processed layers (210 µW m^−1^ K^−2^) reported by Tian et al. The electrical conductivity, Seebeck coefficient and the resulting power factor versus temperature are presented in Figure 6. As mentioned above the variation of the TiS_2_ stoichiometry might be one possible, although speculative, explanation for the systematically higher thermoelectric properties of the printed TiS_2_/HA thin films. However, all the reported HA intercalated TiS_2_ undergo an increase of electrical conductivity and a decrease in Seebeck coefficient. The intercalation of HA is driven by a charge transfer process between amine (slight reducing agent) and TiS_2_ forming bonds with a polar/ionic character due to the interaction between the lone pair of the nitrogen and the 3d orbitals of the Ti atoms: RNH_2_ + TiS_2_ → (RNH_2_)^+^(TiS_2_)^−^. This mechanism implies that HA takes an active role in the modification of the electronic density at the HA/TiS_2_ interface and increase the charge carrier density, thus contributing to a rise in conductivity and decrease in Seebeck coefficient [26]. Hence, we truly speak about a new material formed upon intercalation, also called a hybrid organic-inorganic superlattice of TiS_2_. In those TiS_2_/HA superlattices, the crystallinity seems to play an essential role on the thermoelectric performance; which is a challenge when films are formed by exfoliated nanosheets. The degree of disorder in a semiconductor is expected to smoothen the band edge and decrease the Seebeck coefficient as well as the electrical conductivity. Another reason for the superior thermoelectric performance of our printed film compared to other solution-processed TiS_2_/HA films is the low degree of disordered as reflected by a good crystallinity (see Figure 5). Finally, it is worth to mention here that further increase of the thermoelectric power factor may be still possible as the record value for pristine single crystal TiS_2_ is as high as 3710 µW m^−1^ K^−2^ at 300 K [20].

The printed TiS_2_/HA thin films exhibit a sensitively higher charge carrier concentration of about 2.34 × 10^21^ cm^−3^ than on the previously reported on a polycrystalline TiS_2_/HA film and intercalated TiS_2_ single crystal that are 1.6 × 10^21^ cm^−3^ and 7.59 × 10^20^ cm^−3^, respectively. Using the charge carrier concentration, the intercalation level of the hexylamine into TiS_2_ is estimated as follows. First, it is assumed that each intercalated hexylamine brings an additional charge into TiS_2_. Then, the data published by M. Beaumale et al. [19] et H. Imai et al. [20] are used as references of pristine TiS_2_ with a charge carrier concentration of 1.1 × 10^20^ and 2.8 × 10^20^ cm^−3^, respectively. At the end, the additional charges introduced by the hexylamine intercalation are estimated to be about 2.06 × 10^21^ and 2.23 × 10^21^ cm^−3^ so the resulting intercalation level is about 12–13% in respect of the Ti atoms in the TiS_2_. It means that each eighth Ti atom of the TiS_2_ is affected by the hexylamine intercalation. Similar considerations lead to intercalation levels of about 8 and 3% for TiS_2_/HA obtained by Tian et al. and Wan et al., respectively.

Apart the high charge carrier concentration with a high intercalation level, a relatively high electron mobility of 3.22 cm^2^.V^−1^.s^−1^ at 293 K is observed. It remains lower than the value reported for the intercalated TiS_2_ single crystal (6.41 cm^2^.V^−1^.s^−1^) but higher than the value observed on polycrystalline TiS_2_/HA (2.4 cm^2^.V^−1^.s^−1^). Such values make sense as the electrons are preferentially located on the inorganic TiS_2_ flakes. Finally, the average rate increase of the Seebeck coefficient versus temperature is higher for the hybrid printed layer (34 µV K^−1^ for ΔT = 50 K) than for the hybrid single crystal (17 µV K^−1^ for ΔT = 50 K). These differences in thermoelectric properties could be an indicator of higher effective mass in the hybrid printed TiS_2_/HA films. In conclusions, the enhancement of the thermoelectric properties of the hybrid TiS_2_/HA materials is a result of the fabrication process that leads to higher crystal quality expressed by higher crystal phase purity, larger lateral size of the TiS_2_ flakes and better ordering of the hybrid TiS_2_/HA material.

### 3.5. Influence of the process parameters on the properties of films

Several parameters of the process were tested before finding the optimal conditions. All the experimental details are given in the Supporting Information. First, the impact of the TiS_2_ particle size was investigated. For this purpose, the TiS_2_/HA ink was prepared from a mildly ground home-made TiS_2_ powder (Note S3 and Figure S6, Supporting Information). The reduction of the particle size from few micrometers to below 1 micrometer dramatically impacts the transport properties. Indeed, the electrical conductivity of films is reduced by two orders of magnitude (Table S1, Supporting Information). The hall effect measurements indicate that the initial size of a few micrometers is therefore ideal for the electron transport. Furthermore, the ink formulation process was carried out with different amines such as undecylamine. In all cases the films’ performances were lower than with hexylamine as intercalant, mainly because of a better processability of the latter amine (Note S4 and Figure S7, Supporting Information).

Finally, both of the ink and films aging were investigated. Highest performance is obtained when the film is prepared from a 9000 rpm ink which is only a few hours old. The properties gradually degrade as the ink ages (Note S5 and Figure S8, Supporting Information). In addition, jellification of the ink is observed while it ages. We suspect that a self-ordering between the 2D TiS_2_ flakes and HA molecules leads to the jellification of the ink. It was also concluded that to achieve high power factors the TiS_2_/HA films need to be deposited using non- jellified ink. A similar jellification process was observed on a sonicated MoS_2_ and WS_2_ but the mechanism of the viscosity modification remains unclear [40]. The explanation of this mechanism could be the purpose of another study which should include considerations about the oxidation of the TiS_2_ in the presence of water or oxygen. The mechanism of oxidation was investigated elsewhere by modeling the oxidation pathways of TiS_2_ [41,42]. It was pointed out that such oxidation occurs more easily at the edges of flakes or in the middle of the flakes at sites where sulfur vacancies exist. The influence of the replacement of S atoms by O atoms leads to a bandgap opening which is expected to affect the Seebeck coefficient of the compound and of its intercalated derivatives. In the entire process described in this paper the compounds are exposed to an inert atmosphere ([O_2_] = [H_2_O] = ~1ppm). These conditions might be optimal to avoid the oxidation of TiS_2_ and therefore achieve a high Seebeck coefficient in comparison to the thin films reported elsewhere.

**Figure 7.**
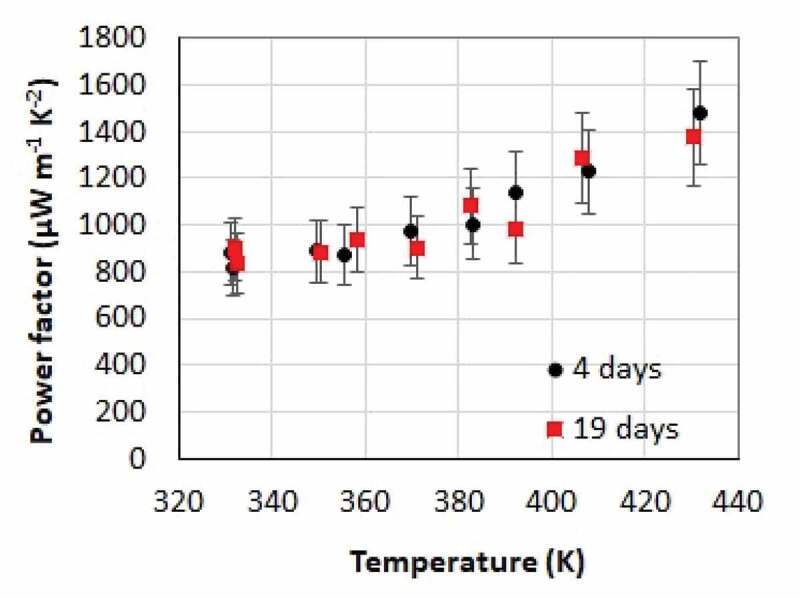
Influence of the films aging on their properties. Temperature dependence of the in-plane power factor of a film 4 days and 19 days after its preparation

The thermoelectric properties of a film were measured 4 days and 19 days after its deposition. The power factor is presented versus temperature in Figure 7. No significant variation is observed. This means that once a film has been deposited, it will remain stable in N_2_ for at least 19 days.

In order to estimate the zT of the TiS_2_/HA films, the measurement of their thermal conductivity is performed by the 3 ω method. Samples are prepared by applying the precursor ink onto Si chips specially designed for this purpose and provided by Linseis [34]. The in-plane thermal conductivity varies from 1.20 W m^−1^ K^−1^ at 330 K to 1.00 W m^−1^ K^−1^ at 430 K for a 2 µm thick film (Figure S9, Supporting Information). The power factor of such a film varies from 800 µW m^−1^ K^−2^ at 330 K to 1000 µW m^−1^ K^−2^ at 430 K. The zT increases from 0.2 to 0.4 when the temperature rises from 330 K to 430 K, which is the highest zT reported for n-type printed thermoelectric materials.

### 3.6. Fabrication of a thermoelectric generator by dispenser printing

**Figure 8.**
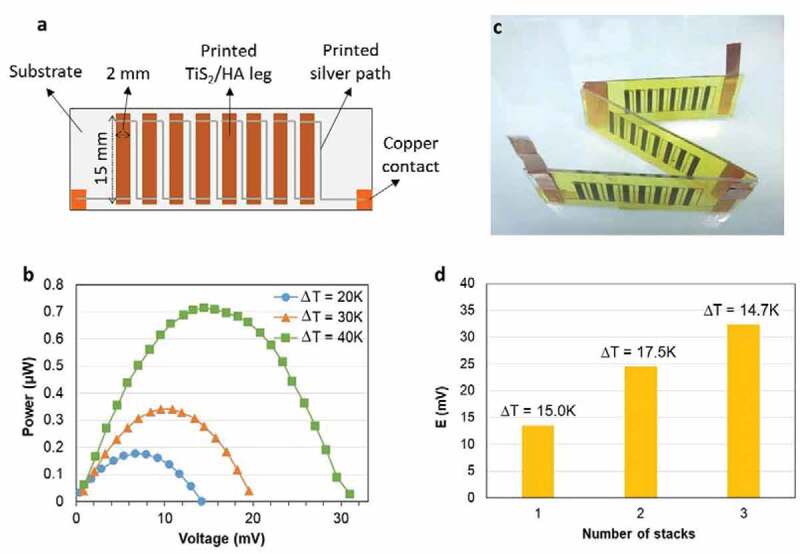
The printed TEG stack and module based on TiS_2_/HA films. (a) Sketch of a TEG stack made of eight TiS_2_/HA legs connected in series. (b) Power output generated by the TEG stack at different temperature gradients. The cold side is at 302 K for the three temperature gradients and the hot side is at 322 K, 332 K and 342 K for the gradients of 20 K, 30 K and 40 K, respectively. (c) Photograph of a TEG module with 3 serial stacks. (d) The voltage generated by TEG stacks connected in series as a function of the number of stacks

Next, the dispenser printer (V-One from Voltera) is used in order to create patterns, i.e. thermoelectric legs, with the TiS_2_/HA inks (Figure 8(a)) either on glass substrate or Kapton® tape. The legs were then connected in series by printing wires of silver paste while copper pieces were used as external contacts for the printed thermoelectric generator stacks (TEG stacks, see Note S6, Figure S10 and Table S2, Supporting Information).

The power output of a TEG stack made of eight connected legs on glass substrate was evaluated (Figure 8(b)). The resistance of the stack is 250 Ω. An open circuit voltage of 30.1 mV is obtained for a temperature gradient of 40 K. The maximum generated power output is 0.713 µW. This outstanding value is almost 3 times above the one reported by Tian et al. for such a temperature gradient. This level of performance would be suitable for low-power wide-area network (LPWAN) [43]. The films have a length of 15 mm, a width of 2 mm and an average thickness of 2.79 µm as shown in Figure S11 (Supporting Information). The power density of the TEG is then calculated at a value of 16.0 W m^−2^, which is also much higher than the values previously reported for the hybrid TiS_2_ superlattices for the same ΔT of 40 K (0.8 W m^−2^).

The individual TEG stacks made of 8 connected legs were connected in series to prepare TEG modules of 1, 2 or 3 stacks. A photograph of module with 3 stacks is shown in Figure 8(c) and the performance of the modules as a function of the number of stacks in Figure 8(d). The voltage generated for a ΔT around 15°C increases gradually as the number of stacks is increased. Note that the measured open-circuit voltage for 2-stack (24 mV) and 3-stack (32 mV) TEG (connected in series) is lower than the calculated voltages (30 mV for 2 stack and 45 mV for 3 stack) due to some defects on the TiS_2_/HA films and quality of contacts TiS_2_/HA-Ag contacts.

Finally, the flexibility and durability tests on TiS_2_/HA legs printed on a Kapton ® tape were carried out. The experimental results showing a very good bending resistance and durability are presented in Supporting Information (Note 7 and Figure S11).

## 4. Conclusions

A fabrication process of hybrid polycrystalline TiS_2_/hexylamine thin films that lead to average power factors of 1100–1460 µW m^−1^ K^−2^ in the 330 K-430 K temperature range has been described. These values exceed the reported performance for hybrid thin films based not only on polycrystalline but also single crystal TiS_2_. It has been shown that these properties are obtained thanks to the proper selection of size of the exfoliated 2D nanoflakes and a better organization of the crystal in the final layered hybrid material as well as high intercalation levels of hexylamine into TiS_2_. They are furthermore strongly influenced by the process parameters such as the quality and particle size of the TiS_2_ powder and the length of the amine chain. In addition, versatile inks were developed which can be directly used in printing processes. Prototype TEG stacks and modules based on n-type printed films were prepared. An outstanding maximum power output of 0.713 µW and power density of 16.0 W m^−2^ for a temperature gradient of 40 K was generated by an individual stack made of eight printed legs connected in series. It is a major achievement to demonstrate that properties even better than those of the corresponding single crystal can be obtained by printing techniques. Such a performance may be useful for the low-power applications e.g. LPWAN that are foreseen for the development of the internet-of-everything.

## Supplementary Material

Supplemental Material
