# Supplementary material for: High-performance flexible thermoelectric modules based on high crystal quality printed TiS2/hexylamine
Source: Sci Technol Adv Mater. 2021 Nov 24;22(1):907–16. doi: 10.1080/14686996.2021.1978802 (PMC8635557; doi:10.1080/14686996.2021.1978802)
Supplement: Supplemental Material [file TSTA_A_1978802_SM2981.docx]

Supporting Information

**High-performance flexible thermoelectric modules based on high crystal quality printed TiS_2_/hexylamine**

*Stéphane Jacob,* Bruno Delatouche, Daniel Péré, Zia Ullah Khan, Marc Jacques Ledoux, Xavier Crispin, and Radoslaw Chmielowski**

**Supplementary Note S1.** Influence of the morphology of the TiS_2_ grains on the quality of TiS_2_/HA films

A commercially available TiS_2_ powder from Sigma Aldrich (Prod. number 333492) and TiS_2_ synthesized in a sealed quartz ampoule were used as precursors for ink formulation and thin film preparation. We have produced about 200 thin films by dr Blake to optimize the condition. The comparison of the thin film microstructures obtained using these different TiS_2_ powders is presented in Figure S1. The thin films obtained from commercially available powders present a disordered layer arrangement and a higher roughness (Figure S1A,B) than the films obtained from TiS_2_ synthesized in quartz ampoule (Figure S1C,D). In addition, some small particles which are embedded in the film can be observed in the films obtained from the commercial powder.

The thermoelectric properties of the films were measured. The electrical conductivity of the material obtained from the commercial TiS_2_ is much lower than that of the film synthesized from the in house TiS_2_. The values vary between 89 S cm^-1^ at 330K and 56.5 S cm^-1^ at 430K, which is almost 10 times below the values observed on samples fabricated from a TiS_2_ synthesized in quartz ampoule (Figure S2A). This low electrical conductivity is explained by the imperfect layered structure revealed by the SEM cross-section analysis. Due to the lower electrical conductivity, the Seebeck coefficient of the film is increased in comparison to the other films (Figure S2B). However, the Power Factor varies between 273 µW m^-1^ K^-2^ at 330K and 472 µW m^-1^ K^-2^ at 430K (Figure S2C).

In conclusion, the films prepared with a TiS_2_ synthesized by heating the Ti and S in an ampoule sealed under vacuum have a much higher power factor than those prepared with the commercially available TiS_2_. This is mainly explained by the differences observed in the starting TiS_2_ powders resulting in layered films with a perfect shape for the home-made TiS_2_ which is not the case for the films prepared with the Sigma Aldrich TiS_2_.


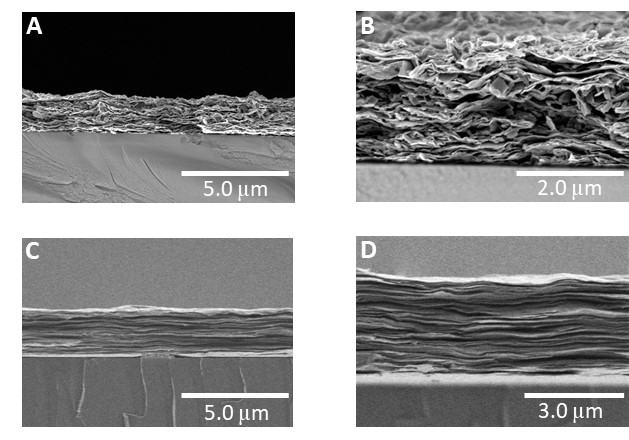


**Figure S1.** Cross-sections of films prepared from a “Sigma Aldrich” TiS_2_/HA ink (A and B at the top) and a home-made TiS_2_/HA ink (C and D at the bottom). A) and C) Magnification 10000. B) Magnification 25000. D) Magnification 15000.


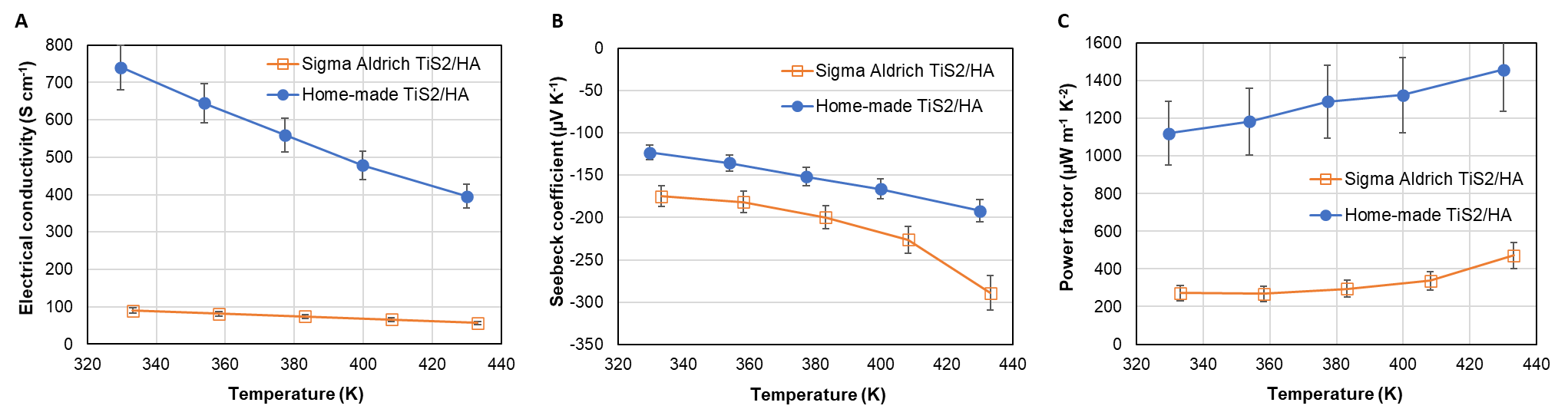


**Figure S2.** In-plane thermoelectric properties of films deposited from “Sigma Aldrich” TiS_2_ and Home-made TiS_2_/HA ink. Temperature dependence of the electrical resistivity (A), the Seebeck coefficient (B) and the power factor (C). ). (The measurements were carried out for a heating and cooling with an accuracy for the Seebeck coefficient of ± 7%, for the electrical conductivity is ± 8%, for the power factor is ± 15%)

**Supplementary Note S2.** Effect of the centrifugation steps on the thin film properties

In the standard process disclosed in this manuscript, the ink used for the deposition of the TiS_2_/hexylamine thin films is obtained by a two-step centrifugation at 1000 rpm and 9000 rpm. To study the role of the first centrifugation a slurry was collected at 1000 rpm and a thin film was prepared.

The cross-sections of both thin films were analyzed by SEM (Figure S3). The film deposited from the 1000 rpm ink contains large particles with a diameter up to ~3 µm. On the contrary, the film deposited from the 9000 rpm has a microstructure composed of well-organized TiS_2_/HA grains. The role of the centrifugation at 1000 rpm is to remove large unintercalated TiS_2_ from the solution and enable the right arrangement of the TiS_2_/HA grains.

The thermoelectric properties of the films are shown in Figure S4. The power factor of the film from the 1000 rpm ink is low. It does not exceed 100 µW m^-1^ K^-2^. Such a low value is mainly due to a very low electrical conductivity varying between 30.1 S cm^-1^ at 330K and 18.7 S cm^-1^ at 408K. The power factor and electrical conductivity of the film from the 9000 rpm ink are much higher. In-plane electron transport is favored by the layered structure, which leads to the much higher electrical conductivity and power factor for the 9000 rpm ink film.


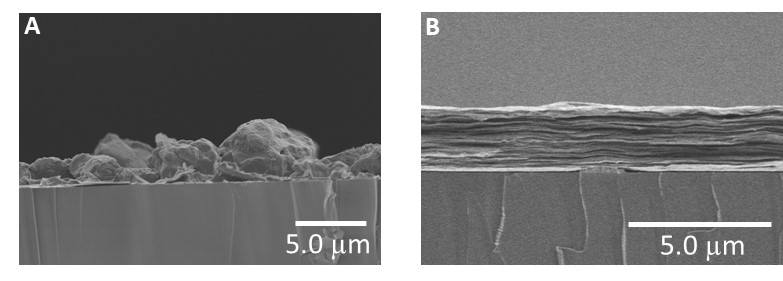


**Figure S3.** Cross-sections of the two series of films. Film from the ink collected after the centrifugation at 1000 rpm (A) and at 9000 rpm (B).


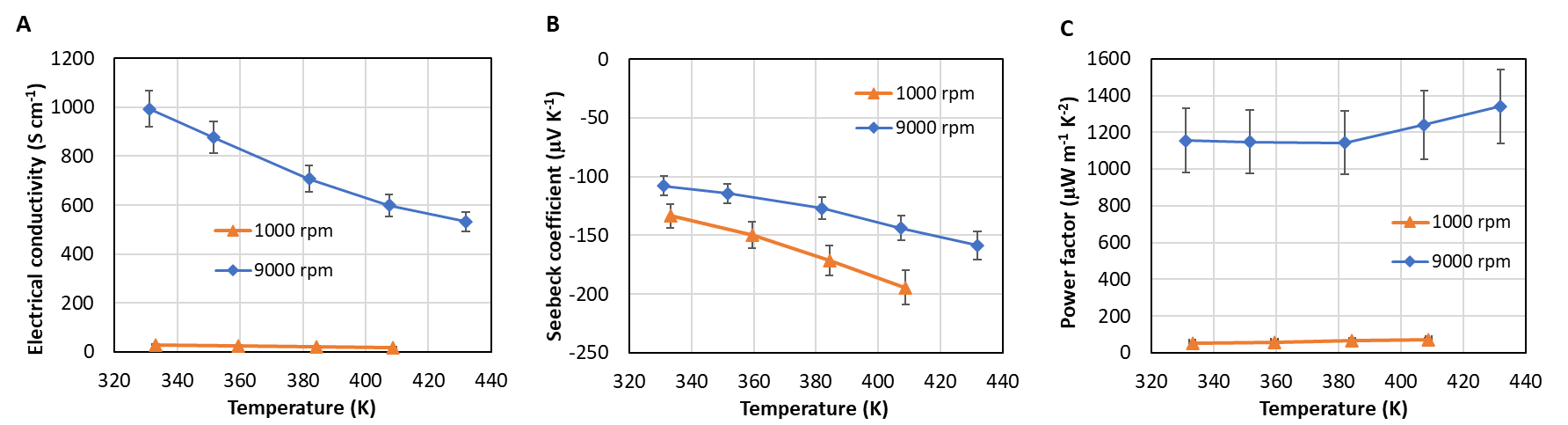


**Figure S4.** In-plane thermoelectric properties of films deposited from the 1000 rpm and 9000 rpm inks. Temperature dependence of the electrical resistivity (A), the Seebeck coefficient (B) and the power factor (C). (The measurements were carried out for a heating and cooling with an accuracy for the Seebeck coefficient of ± 7%, for the electrical conductivity is ± 8%, for the power factor is ± 15%)

**Supplementary Note S3.** Influence of the TiS_2_ particle size

The home-made TiS_2_ was ball-milled to check the influence of the initial particle size on the thermoelectric properties of films prepared according to the new process. The particle size before and after ball-milling is shown in Figure S5. The home-made TiS_2_ shows particles mostly with diameters ranging from several hundred nanometers to a few micrometers. When the material is ball-milled, the particle size is drastically reduced, the diameter being below 1 µm.


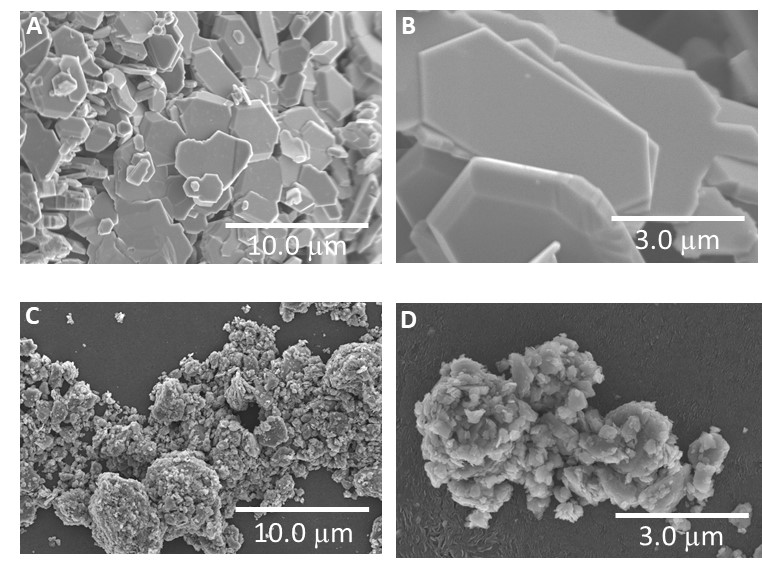


**Figure S5.** SEM pictures of the home-made and ball-milled TiS_2_ powders. Home-made TiS_2_ powder with magnification of 5000 (A) and 15000 (B). Ball-milled TiS_2_ powder with magnification of 5000 (C) and 15000 (D).

**Table S1.** Hall effect measurements on the TiS_2_ films from the home-made and ball-milled TiS_2_ powders.

| **TiS_2_ powder** | **TiS_2_ grain sizes** | **Charge carrier concentration  (cm^-3^)** | **Electrical conductivity  (S cm^-1^)** | **Mobility  (cm^2^ V^-1^ s^-1^)** |
| --- | --- | --- | --- | --- |
| Self-made | Few µms | -2.34+21  ± 1.30E+20 | 1170 ± 1.00 | 3.22 ± 0.10 |
| Ball-milled | <1 µm | -5.86E+19  ± 6.54E+19 | 5.00 ± 0.01 | 5.97 ± 0.25 |

The transport properties of films prepared from the home-made and ball-milled TiS_2_ powders are presented in Table S1. The particle size reduction strongly modifies these properties. The charge carrier concentration is reduced by two orders of magnitude which leads to an electrical conductivity as low as 5.0 S/cm at room temperature. It is therefore important to ensure large size TiS_2_ flakes of a few µm to obtain high electrical conductivity.

**Supplementary Note S4.** TiS_2_/Undecylamine inks and films

Undecylamine (C_11_H_23_NH_2_) is liquid at room temperature. Undecylamine was intercalated in TiS_2_ following the same process as for hexylamine. XRD analysis revealed that the intercalation of such a long chain amine led to an increase of the interlayer distance from 19.6 Å in TiS_2_/HA to 37.9 Å in TiS_2_/UDA. However, in the film prepared by the steps of sonication, 2-step centrifugation, deposition by Blade casting and drying, the interlayer distance was reduced to 10.3 Å. This indicates that the undecylamine molecules are sandwiched parallelly between the TiS_2_ layers as for the dried TiS_2_/HA films. The thermoelectric properties of the TiS_2_/UDA film are shown in Figure S6. The power factor varies between 679 µW m^-1^ K^-2^ at 330K and 849 µW m^-1^ K^-2^ at 430K. These values are ~1.6 times less than those of the TiS_2_/HA films. This decrease in performance is mainly due to a strong decrease of electrical conductivity by a factor 5 which is only in part compensated by the corresponding increase of the Seebeck coefficient. It seems that the in-plane electron movement is favoured in the films intercalated with hexylamine.


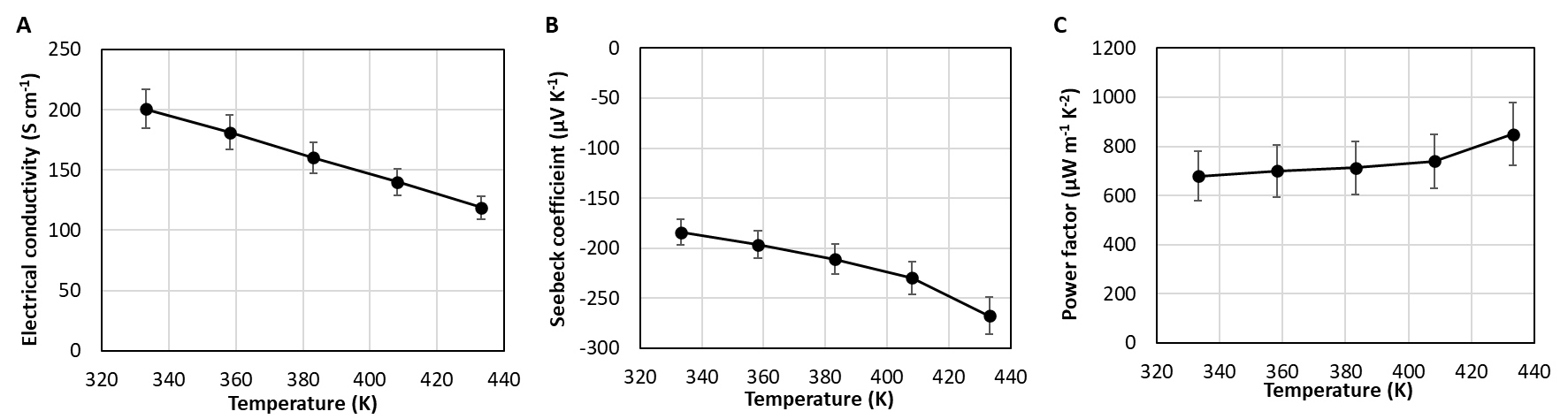


**Figure S6.** In-plane thermoelectric properties of a film deposited from a TiS_2_/Undecylamine ink. Temperature dependence of the electrical resistivity (A), the Seebeck coefficient (B) and the power factor (C).

**Supplementary Note S5.** Aging of the ink

A TiS_2_/HA ink was prepared and films were deposited using this ink 6 hours, 30 hours and 50 hours after its preparation. Their properties are shown in Figure S7. The highest performance was obtained for a film which was prepared with the ink deposited at 6 hours. A high-power factor above 1000 µW m^-1^ K^-2^ was then obtained. The properties degraded gradually as the ink aged, particularly the Seebeck coefficient. The ink deposited at 30 hours led to a film with a PF lowered by more than 40%. XRD analysis was carried out on the different films. The diagrams were similar, indicating that the same phase was observed for all films. The decrease in performance was therefore not due to a modification of the crystal structure. The top surface of a film deposited from an ink prepared several days before the analysis was studied. Some agglomerated particles emerging out of the flat surface of the film were detected. They were not observed on films prepared with a “fresh” ink. This modification of film shape probably led to the decrease in performance. In addition, a modification of the viscosity of the ink was also observed while being aged. Such variations were already reported for a sonication of MoS_2_ and WS_2_ in a round-bottomed flask.^[S1]^ An increase in viscosity was observed which was attributed to a sonication induced polymerization. Such a phenomenon did not occur when using a flat-bottomed flask. Here the sonication was also carried out in a round-bottomed container. The formation of a gel was observed. Finally, these observations showed the existence of some interactions between TiS_2_, HA and NMF in the ink. These interactions were initially beneficial to the film properties, in particular to the Seebeck coefficient which was much higher than in the reported state of the art. In a second stage some induced polymerization might have occurred while the ink was aged. This led to a degradation of the film properties.


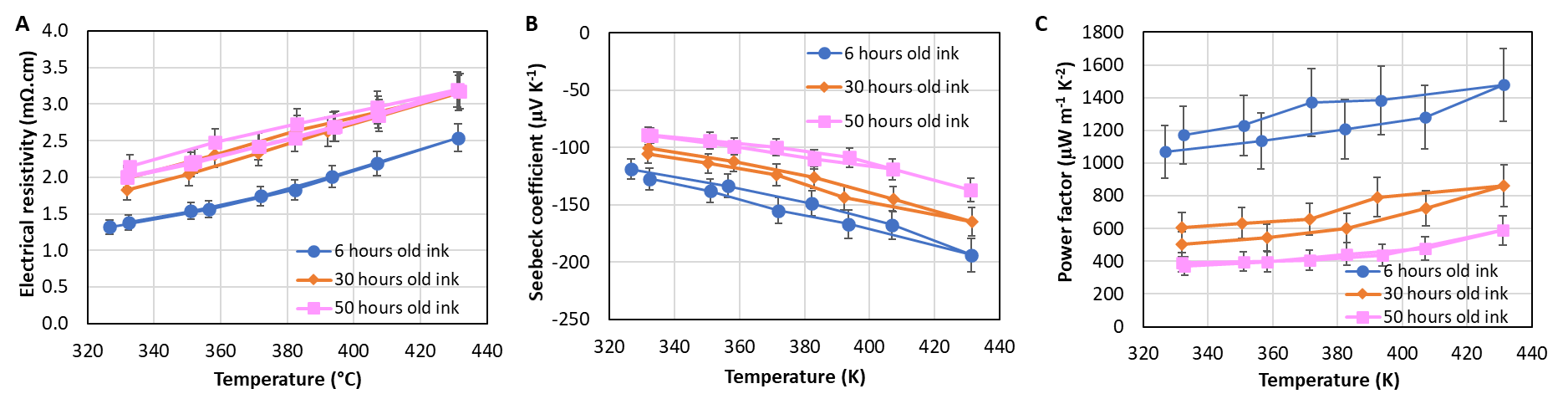


**Figure S7.** In-plane thermoelectric properties of films deposited by blade casting from 6h, 30h or 50h old inks. Temperature dependence of the electrical resistivity (A), the Seebeck coefficient (B) and the power factor (C). (The measurements were carried out for a heating and cooling with an accuracy of 8%, 7% and 15% for the electrical resistivity, the Seebeck and the power factor, respectively)

[S1] Coleman, J. N. *et al*. *Science* **2011**, *331*, 568.


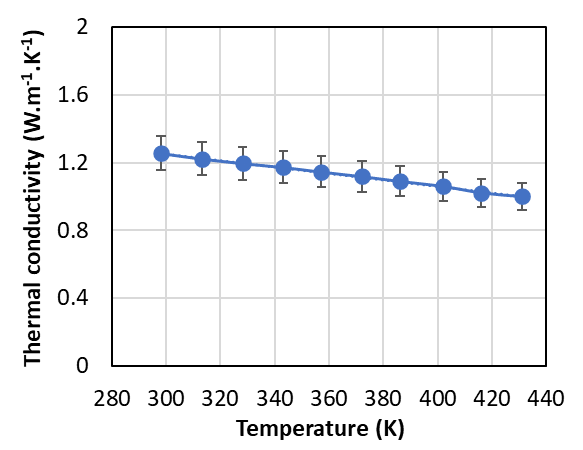


**Figure S8.** Temperature dependence of the in-plane thermal conductivity of a TiS_2_/HA film with a thickness of 2 µm. The ink was deposited on a chip designed for the LFA set-up from Linseis. (The accuracy of the measurement is 8%,

**Supplementary Note S6.** Fabrication of the thermoelectric generator stacks (TEG stacks)

We have produced about 70 samples using the Voltera printer to optimize the deposition conditions. The TEG stacks were prepared by printing eight TiS_2_/HA legs either on glass substrate or flexible Kapton® tape according to the pattern shown in Figure S9A. The legs were then connected in series with printed silver wires. Copper pieces were finally used as external contact which led to the final printed TEG stacks presented in Figure S9B,C.

Four TEG stacks were then prepared on glass substrate and five on Kapton® tape. Table S2 shows the average voltage generated by them for a given temperature gradient and the corresponding Seebeck coefficient. For the stacks on glass substrate, an average generated voltage of 9.7 mV was achieved for a gradient of 12.5°C, which gives a Seebeck coefficient of -98 µV K^-1^ per leg at around 14°C. This value is in the same range as the performance of the films deposited by Blade casting on glass substrate. It confirms therefore that the dispenser printer is a reliable tool to process the TiS_2_/HA films. The property of the stacks on Kapton® tape are slightly weaker with a generated voltage of 9.5 mV for a gradient of 14°C, that gives a Seebeck coefficient of -85 µV K^-1^ per leg at around 14°C.


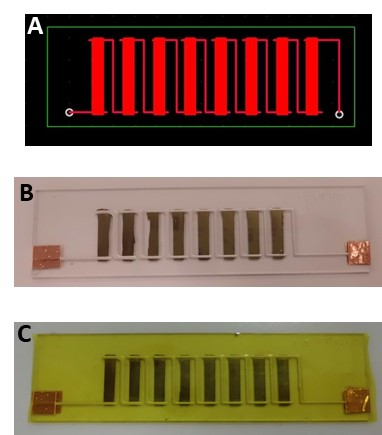


**Figure S9.** The printed thermoelectric generator stacks. A) Pattern targeted for the printing of 8 legs connected in series. The length of a unit leg is 15 mm and its width 2 mm. B) Stack on glass substrate. C) Stack on flexible Kapton® tape.

**Table S2.** **Properties of the nine TEG stacks fabricated either on glass or Kapton® substrate.**

| **Type of substrate** | **E (mV) for 8 legs** | **Gradient (°C)** | **Seebeck coefficient (µV K^-1^) @ ~14°C** |
| --- | --- | --- | --- |
| **Average value for 4 stacks on glass substrate** | -9.7 | 12.5 | -98 |
| **Average value for 5 stacks on Kapton® tape** | -9.5 | 14.0 | -85 |


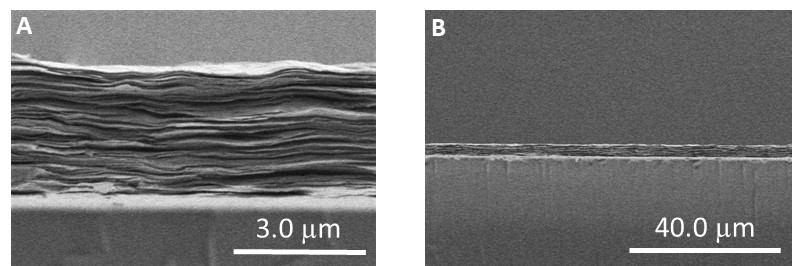


**Figure S10.** SEM picture of the cross-section of a printed TiS_2_/HA leg belonging to the stack with eight connected legs. A) Cross-section with a magnification of 15000. B) Cross-section with a magnification of 1300. The average thickness has been measured at 2.79 µm and was obtained from 32 measurements, 4 measurements for each leg of the stack.

**Supplementary Note S7.** Flexibility test on an encapsulated in a Kapton tape TiS_2_/HA

To check the flexibility of TiS_2_/HA legs and its durability against bending, two experiments were carried out. For both the degradation of the TiS_2_/HA was monitor by the R/Ro ratios where Ro and R are the resistance of the leg at the beginning of the experience and after the bending stress, respectively.

First, an encapsulated in a Kapton ® tape TiS_2_/HA leg was rolled up around drills with a various radius. The tested TiS_2_/HA leg withstands pretty well to the bending test as the degradation of the material starts only when it is rolled on a radius smaller than 5 mm. (Figure S11A) Finally, the sample was destroyed when rolled up around a radius of 2 mm. The geometry of the sample as well as an example of the measurement on a drill of 5 mm of diameter are presented in Figure S11C and D, respectively.

The second check consists on durability test. A new TiS_2_/HA leg was rolled up around a drill of 10 mm of diameter up to 20’000 times. As shown on the Figure S11B, the TiS_2_/HA leg withstands up to 20’000 cycles without any modification of the resistance. The home-made setup used for this experience is presented in Figure S11E.


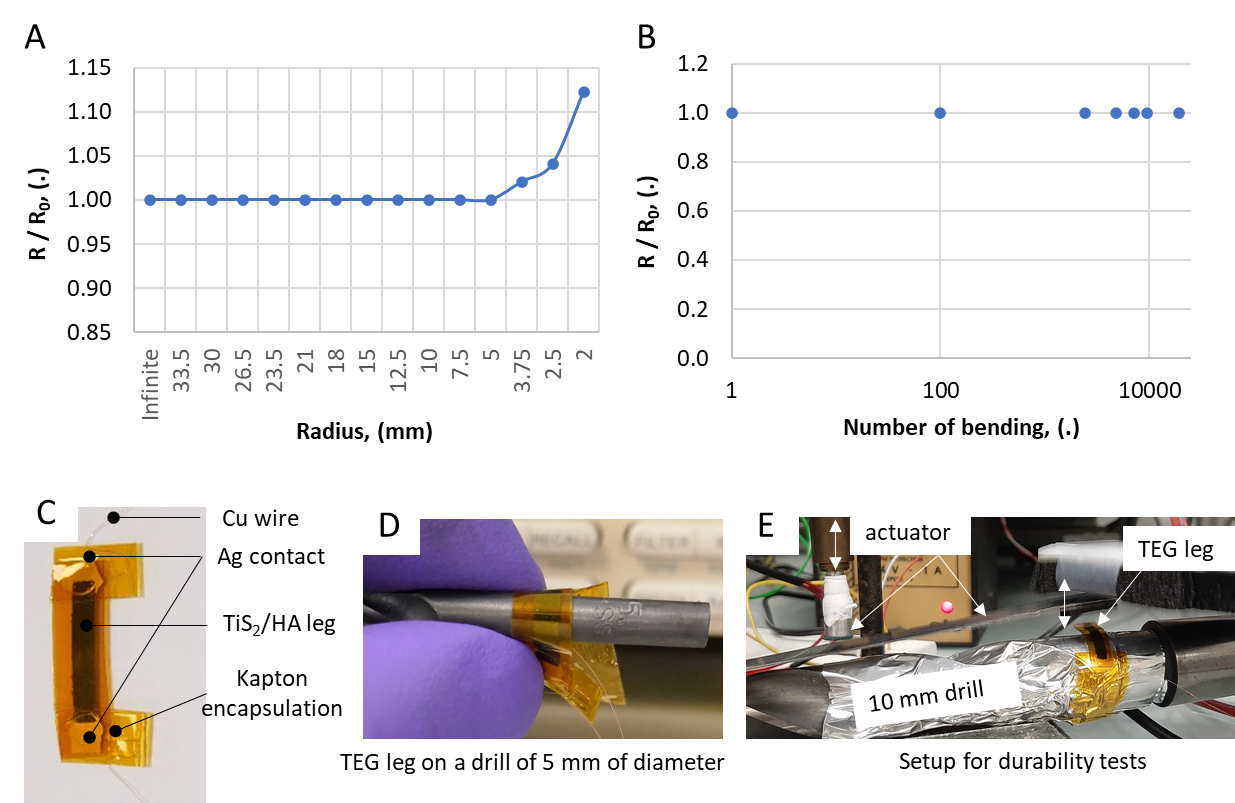


**Figure S11.** Flexibility and durability tests on encapsulated TiS_2_/HA legs. A) The R/R_o_ ratio of a leg when rolled up around drills with various radius. B) The evaluation of the R/R_o_ ratio with the number of bending. The leg is rolled up around a drill of 10 mm of diameter as shown on E. C) An encapsulated TiS_2_/HA leg, D) An example of TiS_2_/HA leg rolled up around a drill of 5 mm of diameter, E) The home-made setup for the durability test.
